# Short‐term actions of epigalocatechin‐3‐gallate in the liver: a mechanistic insight into hypoglycemic and potential toxic effects

**DOI:** 10.1002/2211-5463.70118

**Published:** 2025-09-07

**Authors:** Carla Indianara Bonetti, Bruna Lopes Correia, Francielle Cristina Nakamura Manicardi, Nairana Mithieli de Queiroz Eskuarek Melo, Vanesa de Oliveira Pateis, Jurandir Fernando Comar, Anacharis Babeto de Sá‐Nakanishi, Adelar Bracht, Lívia Bracht

**Affiliations:** ^1^ Post‐Graduate Program in Pharmaceutical Sciences State University of Maringá Maringá Brazil; ^2^ Post‐Graduate Program in Biochemistry State University of Maringá Maringá Brazil; ^3^ Department of Biochemistry State University of Maringá Maringá Brazil

**Keywords:** gluconeogenesis, glycogenolysis, glycolysis, hepatotoxiciy, redox state, β‐oxidation

## Abstract

Epigallocatechin‐3‐gallate (EGCG), the main catechin in green tea, is associated with antidiabetic and anti‐obesity effects, although its acute hepatic actions remain unclear. We investigated short‐term effects of EGCG (10–500 μm) using isolated perfused rat livers and complementary assays in mitochondrial, microsomal, and cytosolic fractions. EGCG markedly inhibited gluconeogenesis from lactate (up to 52%), glycerol (33%), and alanine (47%), while it stimulated glycolysis, glycogenolysis, and oleic acid oxidation (+42% total ketone bodies). Oxygen uptake was stimulated under glycogenolytic and fatty acid oxidizing conditions but inhibited under gluconeogenic conditions. Mechanistic analyses revealed EGCG‐induced mild mitochondrial uncoupling, inhibition of pyruvate carboxylase and glucose‐6‐phosphatase (with no effect on fructose‐1,6‐bisphosphatase) and stimulation of phosphoenolpyruvate carboxykinase. EGCG shifted cytosolic and mitochondrial NADH/NAD^+^ ratios toward oxidation, increased mitochondrial and plasma membrane permeability (LDH leakage from 10 μm), and altered redox‐sensitive fluxes, while the total hepatic ATP content remained unchanged. In summary, EGCG's multifaceted actions suggest that suppression of gluconeogenesis may contribute to its antihyperglycemic effect and the stimulation of fatty acid oxidation to its anti‐obesity action. Finally, EGCG's membrane‐disruptive properties raise concerns about potential hepatotoxicity in compromised livers.

## Graphical abstract

Epigallocatechin‐3‐gallate (EGCG) acutely inhibited gluconeogenesis and enhanced glycolysis, glycogenolysis, and fatty acid oxidation in perfused rat livers. Mechanistic assays revealed mitochondrial uncoupling, inhibition of pyruvate carboxylation and glucose‐6‐phosphatase, shift of NADH/NAD^+^ ratios toward oxidation, and loss of membrane integrity. These multifaceted actions support EGCG’s antidiabetic and anti‐obesity potential but raise concerns about hepatotoxicity under compromised liver conditions.

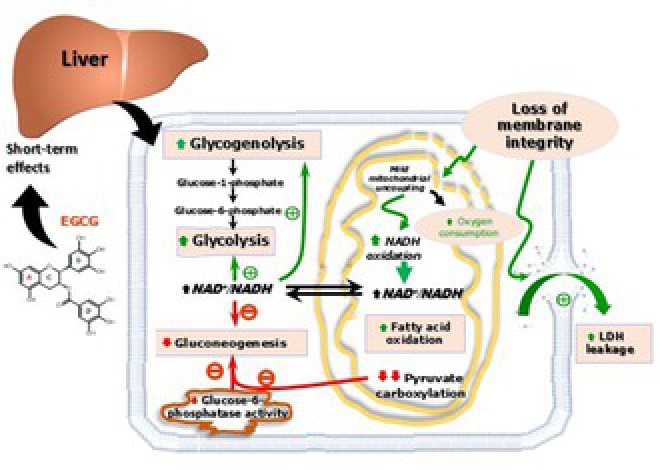

**Abbreviations**
ADP: adenosine diphosphate
AMP: adenosine monophosphate
AMPK: AMP‐activated protein kinase
ATP: adenosine triphosphate
DCF: 2′‐7′‐dichlorofluorescein
DCFA‐DA: 2′‐7′‐dichlorofluorescein diacetate
DCFH: reduced form of 2′‐7′‐dichlorofluorescein
DMSO: dimethylsulfoxide
EDTA: ethylenediamine tetraacetic acid
EGCG: epigallocatechin‐3‐gallate
EGTA: ethyleneglycol tetraacetic acid
FBPase‐1: fructose 1,6‐bisphosphatase
G6Pase: glucose 6‐phosphatase
GAPDH: glyceraldehyde 3‐phosphate dehydrogenase reaction
GDP: guanosine diphosphate
GLUT4: glucose transporter 4
GP: glycogen phosphorylase
GTP: guanosine triphosphate
HEPES: 4‐(2‐hydroxyethyl)piperazine‐1‐ethanesulfonic acid
HPLC: high performance liquid chromatography
IRS‐1: insulin receptor substrate 1
LDH: lactate dehydrogenase
NAD⁺: oxidized nicotinamide adenine dinucleotide
NADH: reduced nicotinamide adenine dinucleotide
PC: pyruvate carboxylase
PEPCK: phosphoenolpyruvate carboxykinase
PGK: 3‐phosphoglycerate kinase
PGM: phosphoglycero mutase
Pi: inorganic phosphate
RC: respiratory control ratio
ROS: reactive oxygen species
TIM: triose phosphate isomerase
TMPD: tetramethylphenylenediamine

Epigalocatechin‐3‐gallate (EGCG), whose chemical structure is shown in Fig. 1, is the most abundant catechin of green tea [1]. It has been claimed that both green tea and pure EGCG could be promising agents in the prevention and treatment of cancer, obesity, diabetes mellitus, and cardiovascular, neural, and hepatic diseases due to their antioxidant, anti‐inflammatory, and antifibrogenic activities [2, 3].

**Fig. 1:**
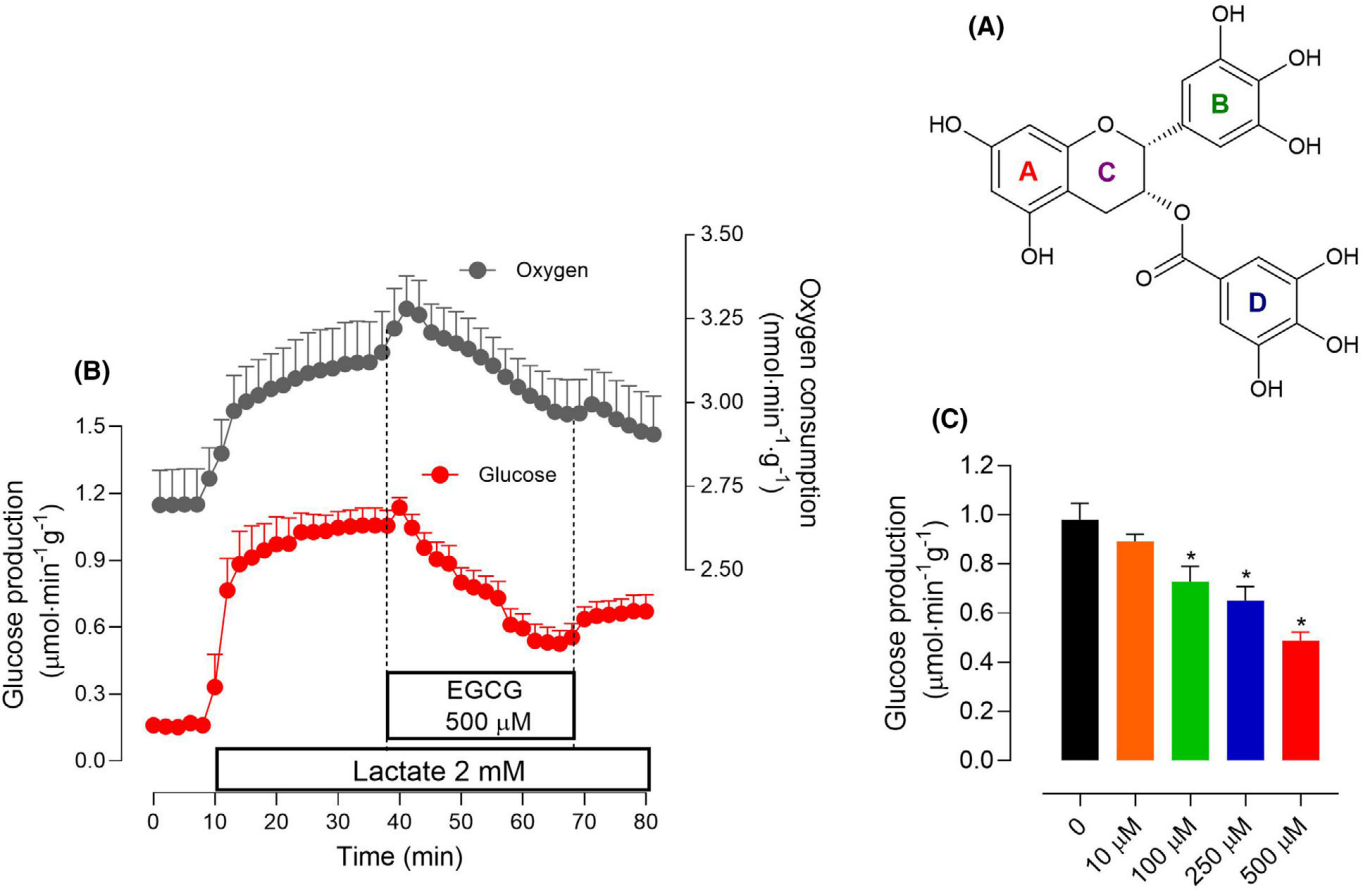
Actions of epigallocatechin‐3‐gallate (EGCG) on lactate gluconeogenesis and the associated increase in oxygen consumption in the isolated perfused liver of fasted rats. (A) Chemical structure of epigallocatechin‐3‐gallate. (B) Time courses of the actions of 2 mm lactate and 500 μm EGCG on glucose production and oxygen consumption. Livers of fasted rats were perfused as described in the Materials and Methods section. Lactate and EGCG were infused as indicated. Samples of the effluent perfusate were collected at 2‐min intervals for assaying their glucose content. Oxygen consumption was monitored polarographically. The data represented are means and SEM of five biologically independent experiments. (C) Concentration dependence of the effect of EGCG on gluconeogenesis after 20‐min infusion (68‐min perfusion time). The same protocol illustrated by panel B was followed for all EGCG concentrations. Means and SEM were represented. The number of biologically independent experiments for each concentration was: *n* = 5, *n* = 4, *n* = 5, *n* = 7 and *n* = 5 for the concentrations 0, 10, 100, 250, and 500 μm, respectively. The mean experimental value measured at 38‐min perfusion time was taken as the control. Asterisks in panel C indicate statistical significance (*P* ≤ 0.05) compared with the control condition, as determined by ANOVA followed by Tukey's *post hoc* test.

Research has shown that EGCG possesses pronounced antidiabetic efficiency demonstrated in preclinical diabetes mellitus models. The actions of EGCG have been proposed to be at least partly mediated by the modulation of the expression of genes involved in the production and utilization of glucose in the liver, as demonstrated *in vitro* [4] (hepatoma H4IIE cells) as well as *in vivo* [4, 5]. Medium‐term exposure of cells to EGCG (up to 8 h) is capable of mimicking the effects of insulin, promoting phosphorylation of tyrosine residues on the insulin receptor and on the substrate 1 of the insulin receptor (IRS‐1) [4]. This event reduces the expression of the genes of glucose‐6‐phosphatase and phosphoenolpyruvate carboxykinase (PEPCK) [4]. Besides these, other long‐term effects (days or weeks of EGCG exposure) were observed, such as the increase in the expression of the glucokinase gene [5] and the diminution of insulin resistance as a consequence of an increase in expression and translocation of the glucose transporter 4 (GLUT4) of the skeletal muscle [6, 7, 8].

In addition to the long‐term or medium‐term effects described above, immediate short‐term effects of EGCG on cellular energetics have been found. In isolated mitochondria and hepatocytes, the substance has been reported to be capable of uncoupling oxidative phosphorylation [9]. If EGCG interferes with mitochondrial energetics, one can speculate that the substance also exerts short‐term effects on metabolic pathways, especially on those ones strictly dependent on mitochondrial energy, such as hepatic gluconeogenesis and several others. This is a question that has not yet been investigated up to now, and it requires a reliable and sensitive experimental system for being approached in a meaningful way. A suitable experimental system for investigating questions of this kind is the isolated perfused rat liver. This system presents a series of advantages when compared to isolated cells. Oxygen uptake can be easily monitored in a continuous way, steady‐state conditions can be induced, and since the organ is largely intact, microcirculation and intercellular trafficking are well preserved [10]. Integrity is important because several metabolic pathways, such as gluconeogenesis and ureagenesis, are compartmentalized and highly sensitive to alterations in cellular integrity [11].

The information about the state‐of‐the‐art in the investigations about EGCG in mammals detailed above prompted us to undertake a systematic study about the short‐term effects of this compound on liver metabolism. The isolated perfused rat liver was used, taking into account the advantages listed in the preceding paragraph. In this study, several biochemical pathways, such as gluconeogenesis from various precursors, glycogenolysis, glycolysis, ketogenesis, and others were quantified in the presence and absence of different concentrations of EGCG. The actions of EGCG on membrane integrity parameters were also evaluated in both the intact liver and isolated mitochondria. Enzymatic assays in subcellular systems, such as mitochondria, microsomes, and cytosol were performed in order to obtain mechanistic information. The results should help in designing and defining aims for the future *in vivo* studies on the pharmacological and toxicological effects of EGCG in both healthy and diseased organisms.

## Materials and Methods

### Materials

Epigallocatechin‐3‐gallate (EGCG), 98.8% purity, was purchased from Chengdu Biopurify Phytochemicals Ltd. (Chengdu, Sichuan, China). Enzymes and coenzymes were obtained from Merck (Darmstadt, Alemanha). All reagent grade chemicals were of analytical purity.

### Animals

Male Wistar rats (50 days old, weighing 180–220 g), obtained from the Central Animal Facility of the State University of Maringá, were used. They were fed with a standard chow diet (Nuvilab®, Quimtia Brasil, Colombo, Brazil) and received tap water *ad libitum*. For the surgical transfer of the liver to the perfusion apparatus and for the procedures of cell fractionation (isolation of mitochondria and microsomes), the animals were previously anesthetized through intraperitoneal injection of ketamine (90 mg·kg^−1^) plus xylazine (9 mg·kg^−1^). Animals were euthanized by exsanguination under anesthesia during the procedures for liver and subcellular fraction isolation. The experimental protocol was previously approved by the Ethics Committee for Animal Experimentation of the State University of Maringá (protocol #6436291021), which follows all recommendations of the Brazilian Law for the use of animals in scientific research.

### Liver perfusion

Isolated rat liver perfusion was done with hemoglobin‐free perfusion fluid in the nonrecirculating mode [12, 13]. After cannulation of the portal and cava veins, the liver was positioned in a specially designed acrylic chamber. The constant perfusate flow, generated by a peristaltic pump (Minipuls 3, Gilson, France), was adjusted between 30 and 32 mL·min^−1^, depending on the liver weight. The perfusion fluid was Krebs/Henseleit‐bicarbonate buffer (pH 7.4) containing 25 mg% bovine serum albumin. The fluid was saturated with a mixture of oxygen and carbon dioxide (95 : 5) by means of a membrane oxygenator with simultaneous temperature adjustment at 37 °C. The traditional composition of the Krebs/Henseleit‐bicarbonate buffer is: 115 mm NaCl, 25 mm NaHCO_3_, 5.8 mm KCl, 1.2 mm Na_2_SO_4_, 1.18 mm MgCl_2_, 1.2 mm NaH_2_PO_4_, and 2.5 mm CaCl_2_. The fluid entered the liver via the portal vein and left the organ via the cava vein. Samples of the effluent perfusion fluid were collected and analyzed for their content in metabolites. Substrates and EGCG were added to and withdrawn from the perfusion fluid at various times according to the experimental protocol. Due to its low water solubility, EGCG was added to the perfusion fluid as a concentrated solution in dimethylsulfoxide (DMSO) for attaining the desired final concentrations (10–500 μm), which are within the limits of its water solubility. The concentration of DMSO in the perfusion fluid was 0.1% (1000 μL·L^−1^). Given that the buffer was infused into the liver at a flow rate of 30–32 mL·min^−1^, the corresponding DMSO delivery rate was approximately 30–32 μL·min^−1^. It is already well documented that the infusion of DMSO at rates of up to 32 μL·min^−1^, a limit that was never surpassed in our experiments, does not affect liver metabolism [14].

### Metabolites assays

The following compounds in the effluent perfusion fluid were analyzed using standard enzymatic procedures: glucose, lactate, pyruvate, β‐hydroxybutyrate, and acetoacetate. Glucose was measured by spectrophotometry (505 nm) using the enzymatic‐colorimetric glucose oxidase method [15]. Lactate and pyruvate were assayed by spectrophotometry (340 nm) using the lactate dehydrogenase reaction [16, 17]. β‐Hydroxybutyrate and acetoacetate were assayed spectrophotometrically using the β‐hydroxybutyrate dehydrogenase reaction [18, 19]. The oxygen concentration in the outflowing perfusate was monitored polarographically by means of a Teflon‐shielded platinum electrode positioned in the acrylic chamber at the exit of the perfusate [12]. The activity of lactate dehydrogenase released into the effluent perfusate was determined using a commercial assay kit (Gold Analisa Diagnóstica Ltda, Belo Horizonte, Brazil). Metabolic rates were calculated from the portal‐cava differences of the fluid flow through the liver and referred to the wet weight of the liver.

The hepatic content of adenine nucleotides (AMP, ADP, and ATP) was measured in liquid nitrogen frozen samples. The frozen tissue was first extracted with 0.4 N perchloric acid. The extract was neutralized with K_2_CO_3_ and the nucleotides were separated and quantified by high‐performance liquid chromatography (HPLC) as already described elsewhere [20].

### Isolation of hepatic mitochondria and microsomes

Mitochondria and microsomes were isolated from fresh rat livers. The peritoneal cavity of rats previously anesthetized as described in sub‐section 2.2 was exposed, the liver removed with scissors and transferred to cold buffer containing 200 mm mannitol, 75 mm sucrose, 0.2 mm ethyleneglycol tetraacetic acid (EGTA), 2 mm tris(hydroxymethyl)amino‐methane (Tris–HCl), pH 7.4, and 50 mg·dL^−1^ bovine serum albumin. The tissue was minced, washed, and homogenized in the same medium in a van Potter‐Elvehjem homogenizer. Mitochondria and microsomes were isolated by differential centrifugation. The first step consisted of a 600 ***g*** centrifugation (10 min) for eliminating nuclei and other debris, followed by a 7000 ***g*** (10 min) centrifugation for precipitating the mitochondria [21]. The supernatant was further centrifuged at 12,400 ***g*** (10 min) for further eliminating debris and unwanted material. Finally, the supernatant was centrifuged at 105 000 ***g*** (60 min) for sedimenting the microsomal fraction [22].

### Mitochondrial respiration

Mitochondrial oxygen consumption was measured by polarography using a Teflon‐shielded platinum electrode [23, 24]. The mitochondria were incubated in a closed oxygraph chamber in a medium (2.0 mL) containing 0.25 m mannitol, 5 mm sodium phosphate, 10 mm KCl, 0.2 mm EDTA, and 10 mm Tris–HCl (pH 7.4). Epigallocatechin‐3‐gallate was added for final concentrations of 20, 50, 100, 150, 250, 500, 750, and 1000 μm. The following substrates were used: succinate (+ rotenone), glutamate + malate, and pyruvate + malate, at final concentrations of 10 mm. The oxygen consumption rates were calculated from the slopes of the recording tracings and expressed as nmol × min^−1^ × (mg protein)^−1^. The respiratory rates were measured under three different conditions: (a) before ADP addition (substrate or basal respiration); (b) just after ADP addition for a final concentration of 0.125 mm (state III respiration); and (c) after cessation of the ADP‐stimulated respiration (state IV). The respiratory control ratio (RC) was calculated as the relation between the state III respiration rate and state IV respiration rate [25].

The NADH oxidase and succinate oxidase activities were assayed polarographically using freeze‐thawing disrupted mitochondria [26]. The incubation medium was buffered with 20 mm Tris–HCl (pH 7.4). The reactions were started by adding the substrates, 1 mm NADH and 1 mm succinate. The same incubation system and procedures were also used for estimating the rate of electron flow through complex IV. TMPD ascorbate was used as the electron donor.

### Mitochondrial ATPase assay

The mitochondrial ATPase activity was measured in structurally intact, coupled and uncoupled, mitochondria as well as in mitochondria disrupted by the freeze and thawing procedure [24]. The intact mitochondria (1.0 mg protein·mL^−1^) were incubated in a medium containing 0.2 m sucrose, 50 mm KCl, 10 mm Tris–HCl buffer (7.4), 0.2 mm EGTA, and 5.0 mm ATP for 20 min at 37 °C, in the absence (coupled) or presence (uncoupled) of 0.2 mm 2,4‐dinitrophenol in a final volume of 0.5 mL. When freeze‐thawing disrupted mitochondria were used, the aqueous incubation medium contained solely 20 mm Tris–HCl buffer (pH 7.4). The onset of the reaction was given by the addition of ATP, and interruption was achieved by the addition of ice‐cold trichloroacetic acid (5%). The activity was quantified by measuring the release of inorganic phosphate according to the original technique of Fiske and Subbarow [27], as detailed by Simões *et al*. [28].

### Assay of enzymes of the gluconeogenic pathway

The activity of glucose 6‐phosphatase (G6Pase) was measured in isolated microsomes by quantifying the release of phosphate, which was measured by means of the traditional Fiske and Subarow technique [26, 27, 29]. The activities of fructose 1,6‐bisphosphatase (FBPase‐1) and phosphoenolpyruvate carboxykinase (PEPCK) were determined in the cytosolic fraction (supernatant after microsome precipitation). Fructose 1,6‐bisphosphatase (FBPase‐1) was measured by quantifying the release of phosphate from the substrate fructose 1,6‐bisphosphate [27, 30]. The PEPCK activity was estimated in a system in which malate dehydrogenase was coupled to the PEPCK reaction. The oxidation of NADH by oxaloacetate formed in the PEPCK reaction was measured spectrophotometrically at 340 nm and taken as the PEPCK activity [31].

The activity of the pyruvate carboxylase in isolated intact mitochondria was evaluated by measuring the incorporation of ^14^C from [^14^C]NaHCO_3_ into components of the tricarboxylic acid cycle [32]. Shortly, EGCG at the concentrations of 10, 100, or 500 μm was added to the incubation medium containing 5 mm pyruvate, 2.5 mm potassium phosphate, 12.5 mm magnesium chloride, 120 mm potassium chloride, 10 mm HEPES buffer (pH 7.5) and 2 mg protein per mL of freshly isolated mitochondria. The reaction was started by the addition of 12 mm [^14^C]NaHCO_3_ (0.25 μCi). After 10 min of incubation at 37 °C under constant agitation, the reaction was ceased by the addition of 0.5 volumes of 2 m perchloric acid. After elimination of the remaining [^14^C]NaHCO_3_, aliquots were taken for counting the incorporated and acid‐stable radioactivity by liquid scintillation spectrometry (TriCarb 2810 TR counter, Perkin Elmer). The scintillation solution for counting ^14^C was composed of toluene/Triton X‐100® (1.5/0.5), 10 g·L^−1^ 1,5‐diphenyloxazole plus 0.4 g·L^−1^ 2,2‐*p*‐phenyl‐bis‐5‐phenyleneoxazole. The incorporated radioactivity was expressed as nmol min^−1^ mg protein^−1^.

### Mitochondrial free radical generation

The generation of mitochondrial reactive oxygen species (ROS, mainly hydrogen peroxide) and other free radicals was estimated by measuring the fluorescence increase due to 2′‐7′‐dichlorofluorescein (DFC) formation from the reduced form of 2′‐7′‐dichlorofluorescein (DFCH). The phenomenon occurs via the oxidation of H_2_O_2_ and other oxygen or nitrogen reactive species in the presence of horseradish peroxidase [33, 34]. In the assay, intact mitochondria (0.8 mg protein) were added to 2 mL of 10 mm HEPES buffer, pH 7.2, containing also 250 mm mannitol, 10 mm succinate, 10 μm rotenone, and 1.36 μm 2′‐7′‐dichlorofluorescein diacetate (DCFA‐DA). Mitochondria contain large activities of esterases and the acetate group of DCFA‐DA is rapidly removed, producing the reduced form DCFH [35]. The reaction was initiated by the addition of horseradish peroxidase (40 μg·mL^−1^) and the fluorescence increase (504 nm for excitation and 529 nm for emission) was read at 1‐min intervals during 10 min. The results were expressed as nmol equivalents min^−1^ (mg protein)^−1^.

### Data analysis

The numerical results in graphs and tables are means ± mean standard errors. Statistical analysis was performed using the GraphPad Prism software (version 8.0). The data were submitted to ANOVA One‐Way analysis with Tukey's *post hoc* testing at the 5% level (*P* ≤ 0.05).

## Results

### Effects of EGCG on hepatic glucose production

**Fig. 2:**
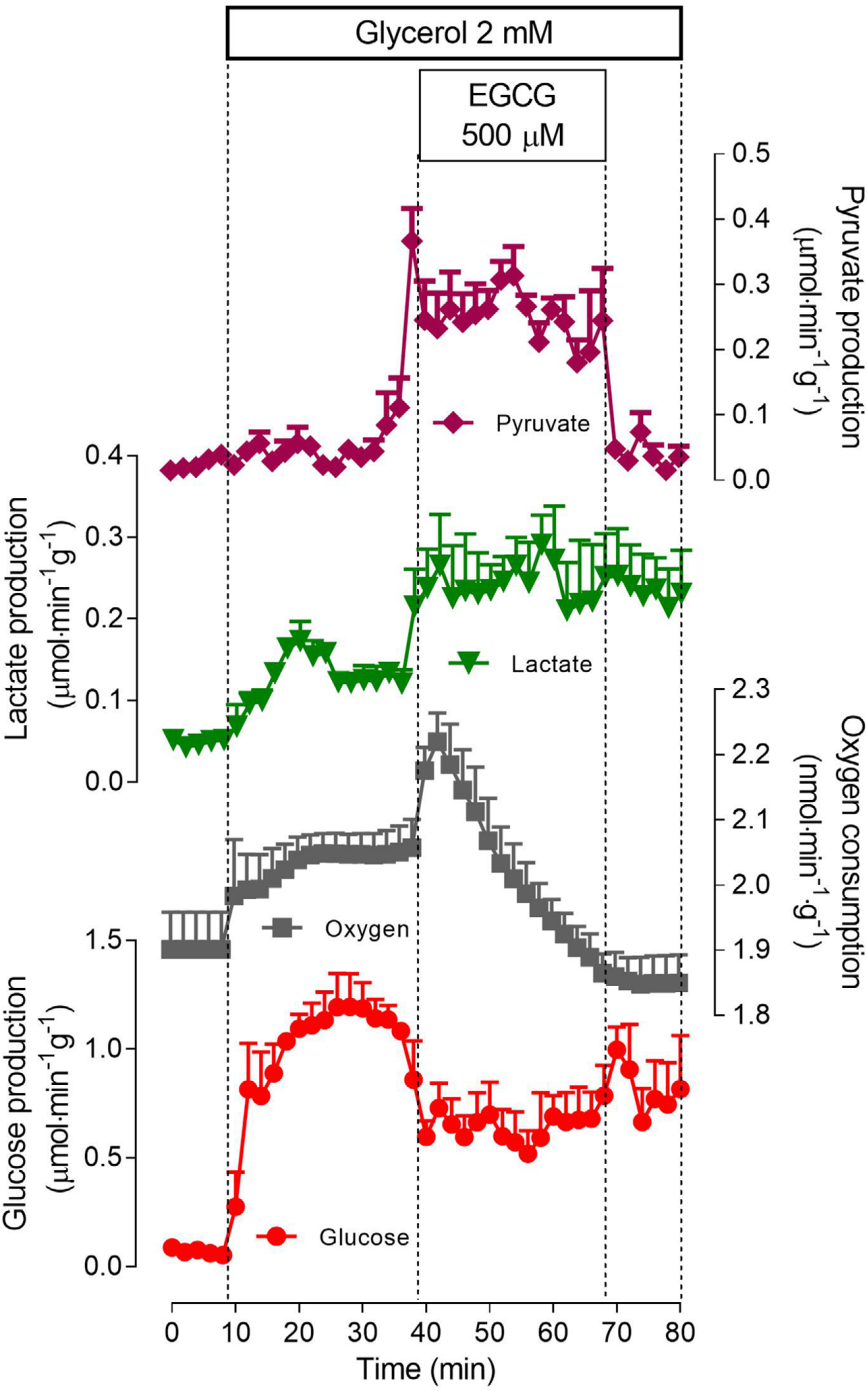
Time course of the effects of 500 μm epigallocatechin‐3‐gallate (EGCG) on 2 mm glycerol metabolism in the perfused rat liver. Livers of fasted rats were perfused as described in the Materials and Methods section. Glycerol and EGCG were infused as indicated. The outflowing perfusate was sampled in 2‐min intervals and analyzed for its contents in glucose, lactate, and pyruvate. Oxygen consumption was monitored polarographically. Means and SEM of three biologically independent experiments were represented.

**Fig. 3:**
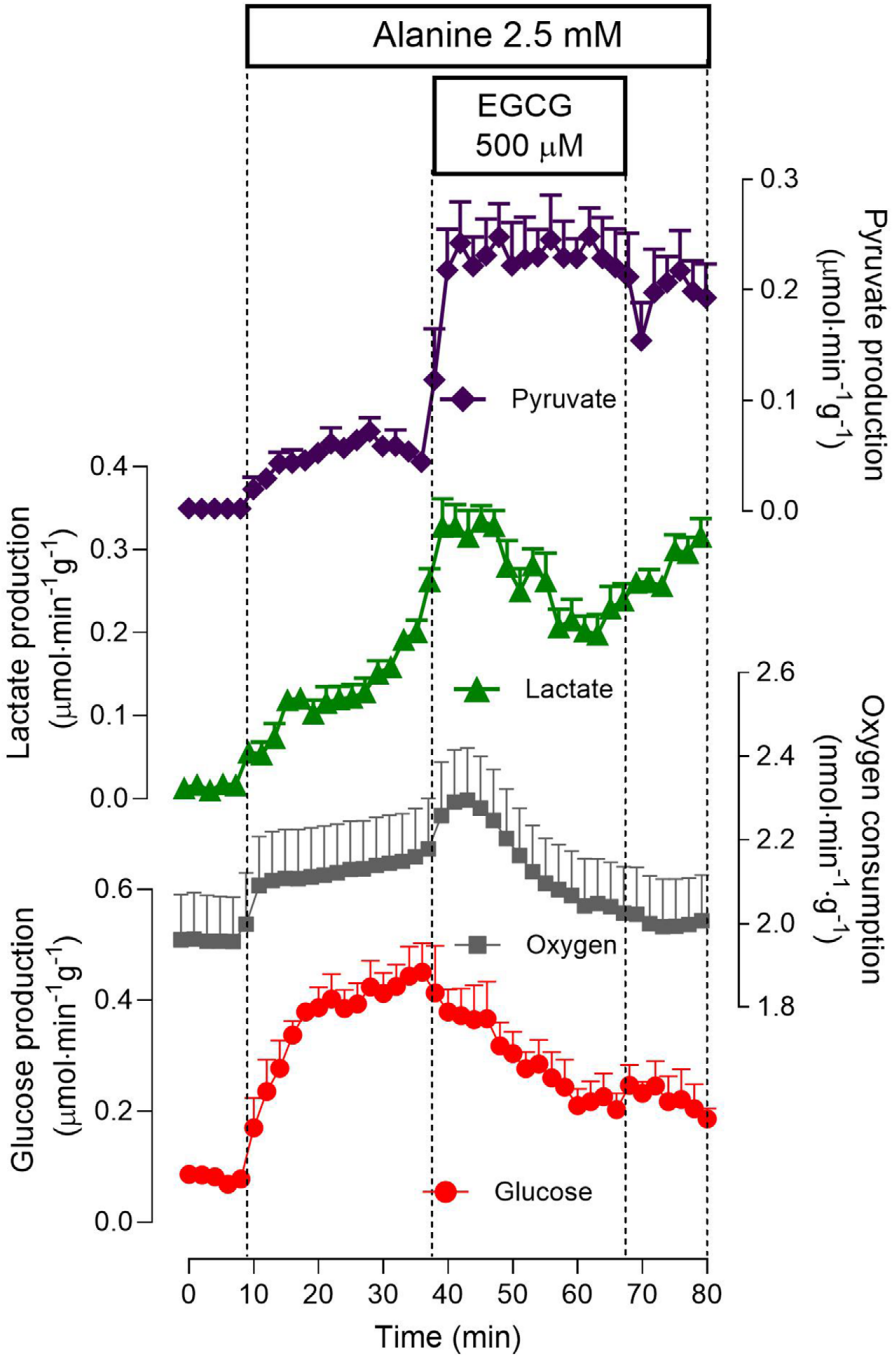
Time course of the effects of 500 μm epigallocatechin‐3‐gallate (EGCG) on 2.5 mm alanine metabolism in the perfused rat liver. Livers of fasted rats were perfused as described in the Materials and Methods section. Alanine and EGCG were infused as indicated. The outflowing perfusate was sampled in 2‐min intervals and analyzed for its contents in glucose, lactate, and pyruvate. Oxygen consumption was monitored polarographically. Means and SEM of three biologically independent experiments were represented.

The actions of EGCG on gluconeogenesis in the liver were investigated using three precursors, namely, lactate, glycerol, and alanine, using fasted animals. Glucose synthesis in the liver is a long pathway that maintains complex relations with and that is influenced by several secondary metabolic routes. For this reason, other associated parameters, such as oxygen consumption and lactate/pyruvate production, were measured simultaneously to enlarge the interpretative field. The results of these experiments can be seen in Figs 1, 2, 3.

Figure 1 illustrates the results obtained when lactate was used as the gluconeogenic substrate. Panel B shows the time course of the changes in glucose production and oxygen consumption due to the infusion of lactate and 500 μm EGCG. This graph, besides showing the results, also illustrates the experimental protocol that was used. The liver was initially perfused with substrate‐free Krebs/Henseleit‐bicarbonate buffer during the first 10 min. Glucose output by the liver during this period was very small because the glycogen stores of livers from fasted rats are minimal and the perfusion fluid does not contain gluconeogenic substrates. The infusion of lactate at 10‐min perfusion time caused a progressive increase in glucose output as well as in oxygen consumption. After 25 min, approximately, new steady‐states were reached. Energy for glucose synthesis under this situation comes predominantly from the oxidation of endogenous fatty acids [36]. The introduction of EGCG at 38 min elicited a gradual diminution of glucose production, reaching 52% inhibition at the end of the infusion period (68‐min perfusion time). Oxygen consumption was transiently stimulated by EGCG, but inhibition occurred almost in parallel with the inhibition of glucose production. Actually, inhibition proceeded in a way that was proportional to the elapsed time, especially that of glucose production, suggesting that the effects were still not complete when the infusion of EGCG was stopped. After ceasing the EGCG infusion, there was a discrete increase in glucose production, but with no signs of restoring the initial levels in a short time. The experiment shown in panel A was repeated with other EGCG concentrations. The rates of glucose production before and after 30 min of EGCG infusion were evaluated and the results are shown in panel C. The effects of EGCG are clearly concentration dependent and they apparently start at the concentration of 10 μm, even though no statistical significance at the 5% level was found at this concentration.

The effects of 500 μm EGCG on glycerol metabolism, a physiologically important gluconeogenic substrate during fasting, are illustrated by Fig. 2. Glycerol undergoes anabolic and catabolic reactions when infused into the liver. Glucose production was 33% inhibited by 500 μm EGCG. Contrary to what happened with lactate, the effect developed rapidly and seemed to have been completed during the infusion time period (30 min). Transformation of glycerol into pyruvate and lactate, on the contrary, was stimulated by EGCG. The effect was more pronounced on pyruvate production. In consequence, the lactate to pyruvate ratio was decreased by EGCG, from approximately 3.0 to 1.0. Oxygen consumption was again at first transiently stimulated, inhibition taking place thereafter in a progressive way and reaching final levels well under the basal rate. Excepting pyruvate production, all other effects did not present reversibility, at least not within the short time of 12 min.

The effects of 500 μm EGCG on alanine metabolism are shown in Fig. 3. Alanine is also an important carbon source for glucose synthesis in the liver, and its deamination leads to the production of lactate, pyruvate, and ammonia, which is an important precursor in nitrogen metabolism. Figure 3 shows inhibition patterns of glucose production from alanine and of oxygen consumption that are very similar to those observed when lactate was the carbon source. The inhibition of glucose production reached 47% at the end of the EGCG infusion period. Lactate and pyruvate production were increased instead by EGCG. The increment in lactate production showed an initial burst and a declining tendency thereafter as the EGCG infusion progressed. The lactate to pyruvate ratio was approximately 3.0 before the EGCG infusion and decreased to 1.0 at the end of the infusion.

### Effects of EGCG on glycogen catabolism

**Fig. 4:**
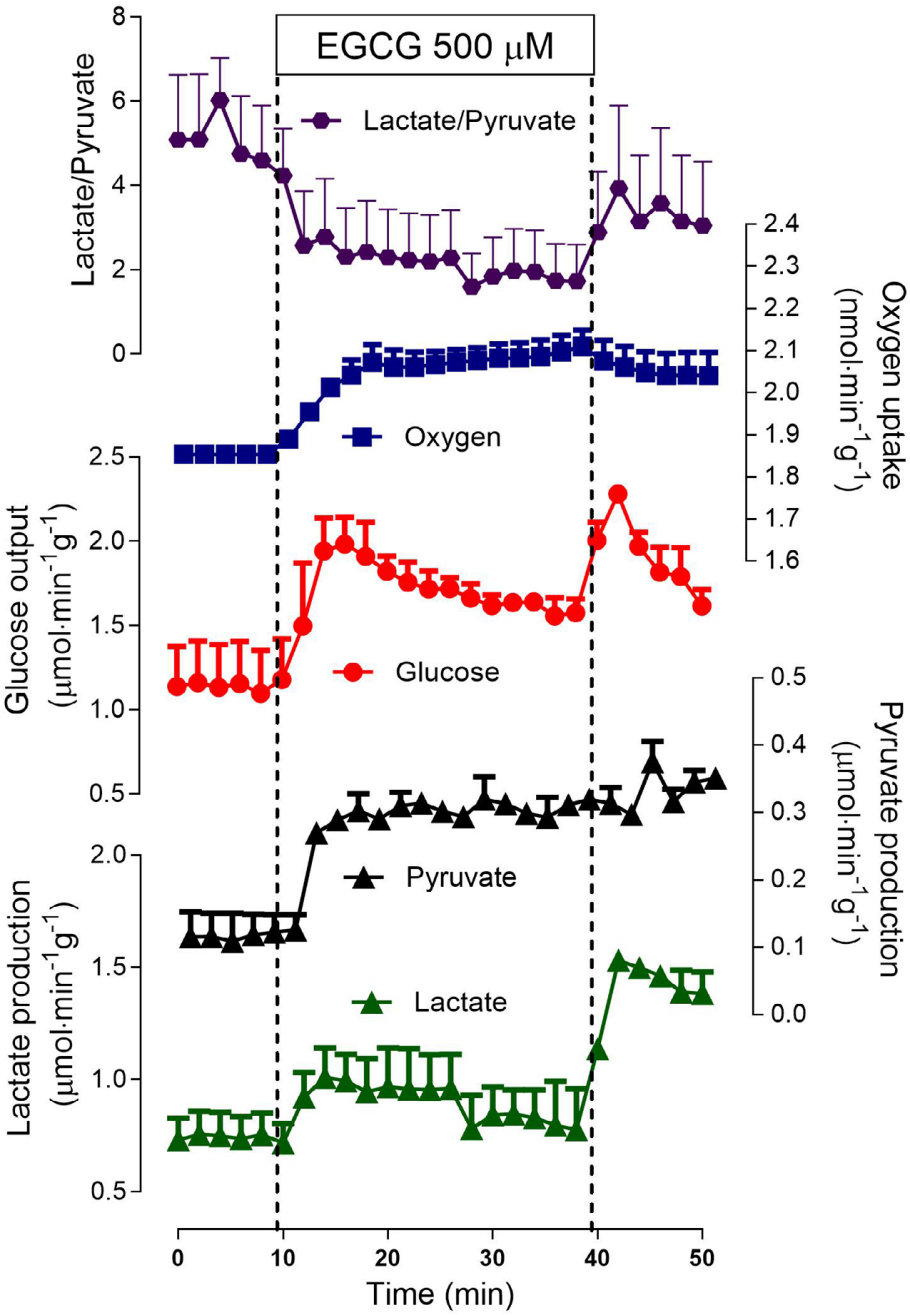
Time course of the actions of 500 μm epigallocatechin‐3‐gallate (EGCG) on glycogen catabolism and oxygen consumption. Livers of fed rats were perfused with substrate‐free perfusion fluid as described in the Materials and Methods section. EGCG was infused as indicated. The outflowing perfusate was sampled in 2‐min intervals and analyzed for its contents in glucose, lactate, and pyruvate. Oxygen consumption was monitored polarographically. Means and SEM of four biologically independent experiments were represented.

**Fig. 5:**
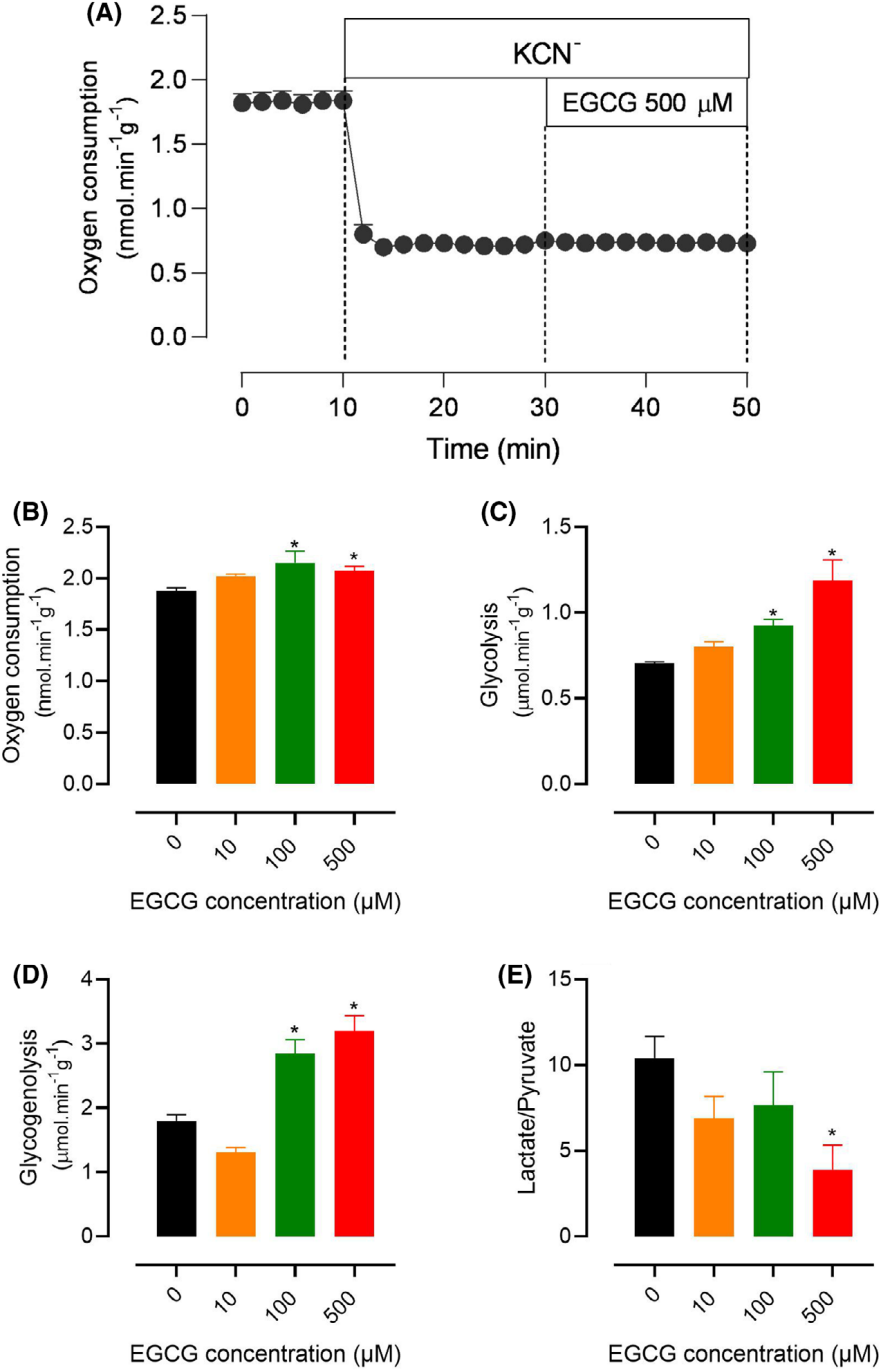
Influence of 500 μm epigallocatechin‐3‐gallate (EGCG) on oxygen consumption in livers from fed rats after blocking the mitochondrial respiratory chain with cyanide (panel A) and concentration dependences of the effects of EGCG on oxygen consumption (panel B), glycolysis (panel C), glycogenolysis (panel D), and the lactate/pyruvate ratio (panel E). Livers of fed rats were perfused with substrate‐free perfusion fluid as described in the Materials and Methods section. Panel A: 2 mm KCN (2 mm) was infused alone for 20 min and cumulatively with 500 μm EGCG for the next 20 min, as indicated. Oxygen consumption was monitored polarographically. The data represented in panel A are the means and SEM of 3 biologically independent experiments. Panels B–E: the experiments were performed according to the protocol illustrated by Fig. 4, but with various EGCG concentrations. Glycolysis was calculated as lactate + pyruvate productions. Glycogenolysis as glucose production + (glycolysis)/2. Values of the variables before the EGCG infusion (8‐min perfusion time; absence of EGCG) and at the end of the EGCG infusion (38‐min perfusion time) were represented. All values in panels B–E are the means and SEM of 4 biologically independent experiments for each concentration. Asterisks indicate statistical significance (*P* ≤ 0.05) with respect to the control condition, as determined by ANOVA followed by Tukey's *post ho*c test.

The liver of fed rats is characterized by its much higher levels of glycogen providing, thus, the opportunity of quantifying glycogenolysis and glycolysis. The results of experiments in which the actions of three EGCG concentrations (10, 100, and 500 μm) on the production of indicators (glucose, lactate, and pyruvate productions) of glycogen catabolism were investigated are shown in Figs 4 and 5. The time course of the actions of 500 μm de EGCG is shown in Fig. 4, which also illustrates the experimental protocol. No substrates were infused, and sampling of the outflowing perfusate was initiated after oxygen consumption stabilization. After 10 min, the infusion of EGCG started and continued for the next 30 min. After this time, perfusion was still maintained for 12 min. EGCG increased all parameters that were measured: oxygen consumption, glucose output, lactate production, and pyruvate production. Notably, the interruption of EGCG infusion at 38‐min perfusion time resulted in further increases in lactate and glucose output. The effects on oxygen consumption and pyruvate production, on the contrary, remained at their higher levels after cessation of EGCG infusion. The lactate to pyruvate ratio, which is known to reflect the cytosolic NADH/NAD^+^ ratio [37], was clearly decreased during the infusion period of EGCG, with a tendency of returning to the previous values when the infusion was stopped.

It is worth at this point to obtain more information about the action of EGCG on oxygen consumption, which was inhibitory in livers from fasted rats (Figs 1, 2, 3), but stimulatory in livers from fed rats. The latter could be of mitochondrial origin, but it could equally result from stimulation of mixed function oxidation via cytochrome P450. In order to decide among these two possibilities, the mitochondrial respiratory chain was blocked by 2 mm potassium cyanide. If mixed function oxidation is increased by EGCG under these conditions, oxygen consumption by the liver should still be stimulated by the latter compound. Panel A in Fig. 5 shows how extensively cyanide blocks respiration of the liver when it is infused. The subsequent cumulative infusion of EGCG did not cause any stimulation in oxygen consumption. These results very strongly indicate that oxygen consumption stimulation by EGCG occurs in mitochondria rather than in the mixed function oxidases dependent on cytochrome P450.

Figure 5 shows also the concentration dependences of the modifications caused by EGCG on glycogen catabolism and oxygen consumption. These data were obtained using the same experimental protocol illustrated by Fig. 4, but with the infusion of EGCG at two other different concentrations, namely, 10 and 100 μm. The tendency for increasing oxygen consumption was already present at the concentration of 10 μm, although significance was given at the concentrations of 100 and 500 μm (panel B). Glycogen catabolism in Fig. 5 was represented in terms of glycolysis (lactate + pyruvate productions) and total glycogenolysis (glucose production + glycolysis/2) in panels C and D, respectively, and in terms of the lactate to pyruvate ratio in panel D. EGCG stimulated glycogenolysis and glycolysis, but inhibited the lactate/pyruvate ratio (which reflects cytosolic NADH/NAD^+^ ratio).

### Effects of EGCG on oleic acid catabolism

**Fig. 6:**
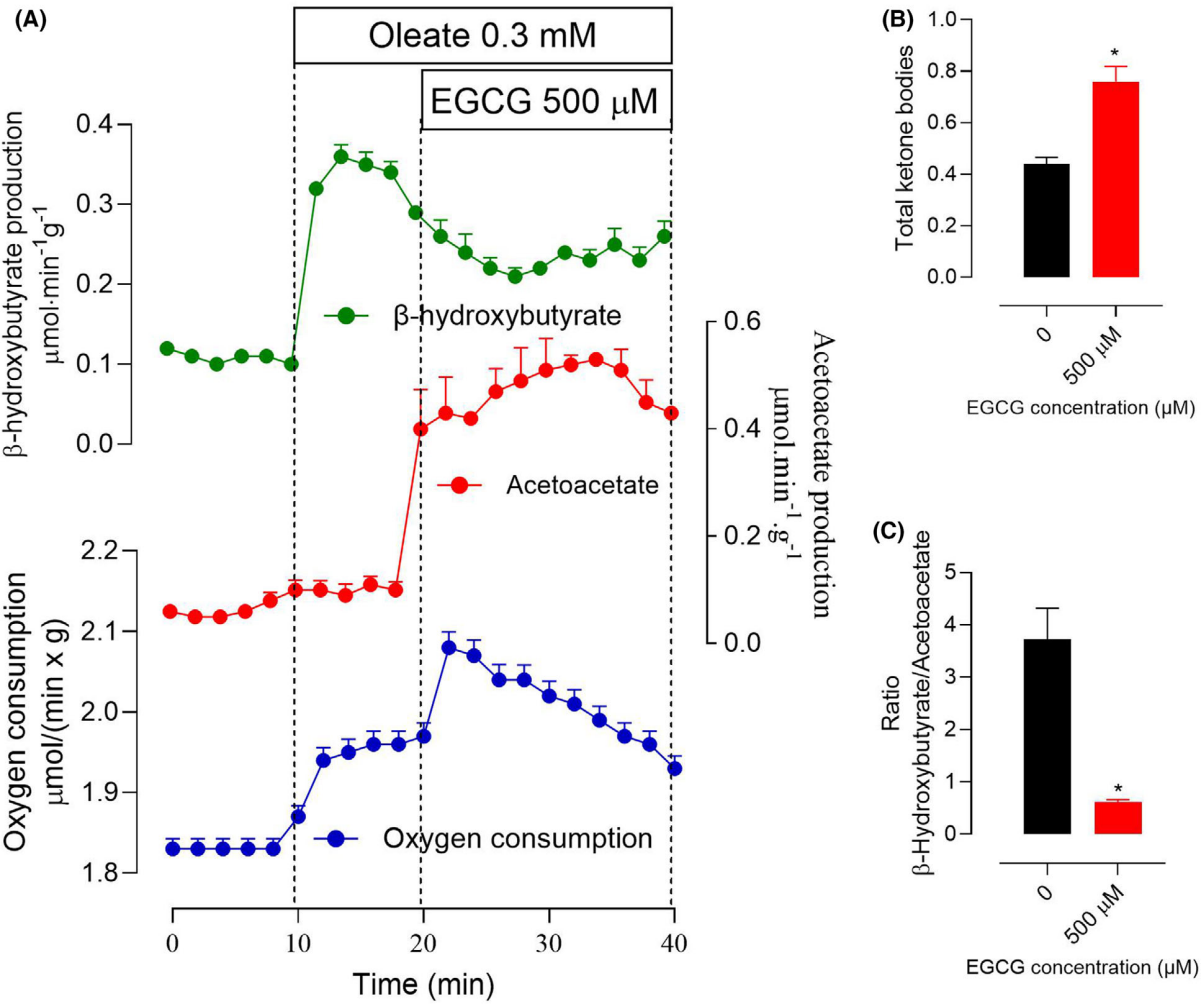
Actions of epigallocatechin‐3‐gallate (EGCG) on the increments in ketogenesis and oxygen consumption caused by oleic acid infusion. Livers of fed rats were perfused as described in the Materials and Methods section. Panel A shows the time course of the experiments in which 0.3 mm oleate was initially infused alone during 10 min and thereafter cumulatively with 500 μm EGCG, as indicated. The outflowing perfusate was sampled in 2‐min intervals and its content in β‐hydroxybutyrate and acetoacetate was assayed. Oxygen consumption was monitored polarographically. Panel B compares the total production of ketone bodies (β‐hydroxybutyrate and acetoacetate) before (18‐min perfusion time) and after (40‐min perfusion time) the introduction of EGCG. Panel C compares the total β‐hydroxybutyrate to acetoacetate ratios before (18‐min perfusion time) and after the introduction of EGCG (40‐min perfusion time). All experimental points are means and SEM of biologically independent experiments (*n* = 3). Asterisks indicate statistically significant differences (*P* ≤ 0.05) between the absence and presence of EGCG, as determined by ANOVA followed by Tukey's *post hoc* test.

Oxygen uptake in the mitochondrial respiratory chain in substrate‐free perfused livers occurs mainly at the expense of endogenous fatty acids oxidation [36]. Since EGCG stimulates oxygen consumption in substrate‐free perfused livers, it is of interest to know whether the compound also stimulates catabolism of exogenous fatty acids. To address this question, oleic acid—a monounsaturated fatty acid—was used as a precursor, and oxidation was evaluated by measuring oxygen consumption and ketone bodies production in the liver of fasted rats. In this condition, it is commonly agreed that formation of ketone bodies is the major metabolic fate of acetyl‐CoA produced by fatty acid oxidation in the liver [38]. The results that were obtained are shown in Fig. 6. The infusion of oleic acid was accompanied by increases in oxygen consumption and ketone bodies production, that is, β‐hydroxybutyrate and acetoacetate, with predominance of the first one. These variables can be regarded as indicators for the catabolism of oleic acid. The introduction of 500 μm EGCG caused a transient stimulation of oxygen consumption, a small diminution in β‐hydroxybutyrate, and a pronounced increase in acetoacetate productions (panel A). By analyzing the total increase in ketone bodies production EGCG, that is, β‐hydroxybutyrate + acetoacetate (panel B), it can be seen that it reached 42%. The β‐hydroxybutyrate to acetoacetate ratio, on the contrary, was substantially decreased (panel C). The mitochondrial β‐hydroxybutyrate dehydrogenase is an enzyme that operates under near‐equilibrium conditions, and it rapidly interconverts β‐hydroxybutyrate and acetoacetate according to the mitochondrial NADH/NAD^+^ ratio. Changes in the hydroxybutyrate to acetoacetate ratio, thus, reflect changes in the mitochondrial NADH to NAD^+^ ratio [37].

### Effects of EGCG on mitochondrial respiration

**Fig. 7:**
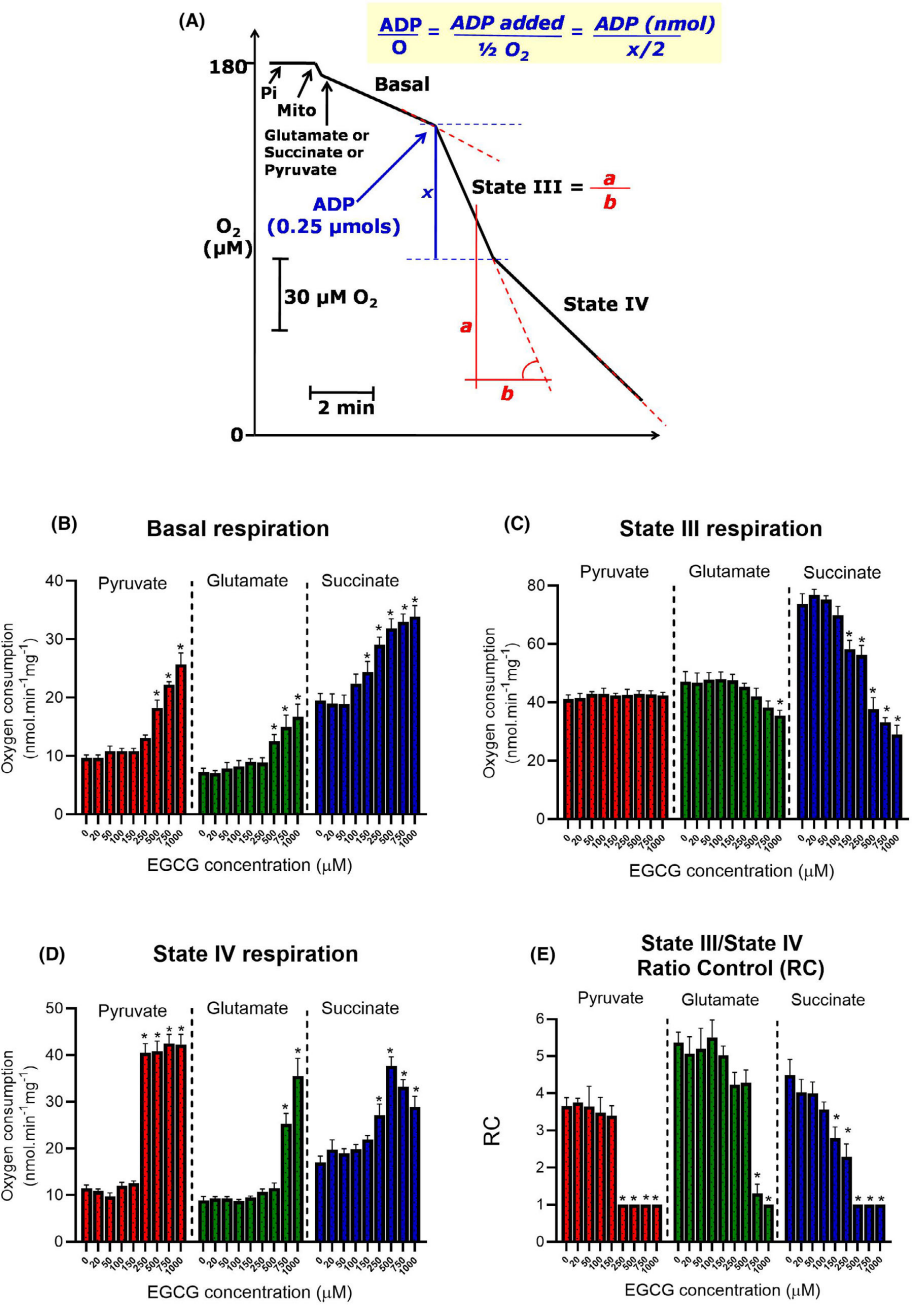
Epigallocatechin‐3‐gallate (EGCG) and the respiratory activity of isolated rat liver mitochondria. Mitochondria (0.5–1.0 mg·mL^−1^) were added to the reaction medium in the closed oxygraph. EGCG was dissolved in dimethylsulfoxide (DMSO) and aliquots were added to the reaction medium for final concentrations of 20–1000 μm. Controls containing just DMSO were also run (zero EGCG). Respiration was started by the addition of one of the following combinations: pyruvate + l‐malate, l‐glutamate + l‐malate, and succinate + rotenone. The oxygen concentration was followed polarographically for 5 min. After this time, 0.25–0.5 nmol ADP was added. The rates of oxygen consumption were computed from the slopes of the polarographic recordings, as illustrated in panel A. The respiratory control ratio (RC) was calculated as (state III rate)/(state IV rate). Each bar (Panels B–D) represents mean ± SEM of *n* = 5, *n* = 7, and *n* = 5 independent experiments, respectively. Asterisks indicate statistically significant differences when compared to the controls (*P* ≤ 0.05), as determined by ANOVA followed by Tukey's *post hoc* test.

The strong evidence obtained in the experiments shown in Fig. 5 that the hepatic changes in oxygen consumption caused by EGCG occur in the mitochondria prompted us to investigate a possible direct action on these organelles. The results are shown in Fig. 7. Panel A illustrates the experimental protocol, the typical behavior of isolated mitochondria incubated in a closed oxygraph chamber and the way by which the parameters represented in the graphs of panels B, C, D, and E were evaluated [23, 39]. Three substrates or substrate combinations were used: pyruvate + l‐malate, l‐glutamate + l‐malate, and succinate + rotenone. In general, it can be said that EGCG increased basal and state IV respiration with all substrates. State III respiration, however, was inhibited with glutamate and succinate as substrates, but not when pyruvate was the substrate. The respiratory control ratio, which is a measure of the capacity of returning to a state of reduced oxygen consumption after the acceleration caused by ADP phosphorylation, was diminished by EGCG, indicative of a certain degree of uncoupling. All these effects of EGCG tended to occur only at concentrations equal to or above 500 μm.

### Effects on mitochondrial membrane‐bound enzymatic activities

**Fig. 8:**
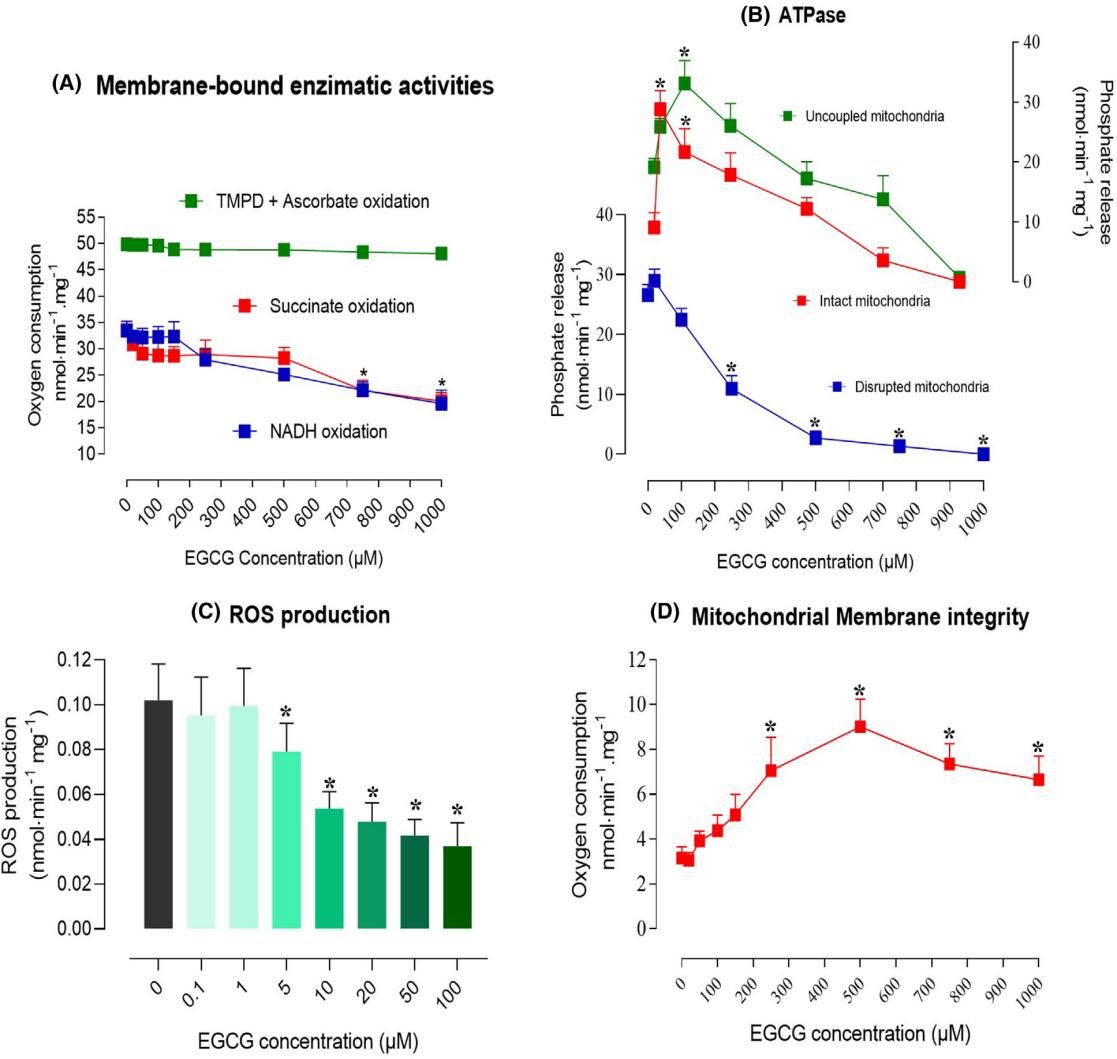
Effects of epigallocatechin‐3‐gallate (EGCG) on mitochondrial membrane linked enzyme activities, ATPase activity, reactive oxygen species (ROS) production and membrane integrity. (A) Membrane linked oxidase activities were measured in freeze‐thawing disrupted mitochondria. The mitochondria (0.5–1.0 mg·mL^−1^) were added to the reaction medium in the closed oxygraph chamber. The reaction was initiated by the addition of NADH, succinato + rotenone or TMPD/ascorbato and oxygen consumption was recorded polarographically. Each experimental point represented in panel A is the mean ± SEM of seven independent experiments. (B) The ATPase activities of intact mitochondria (1.0 mg·mL^−1^) in the presence or absence of 2,4‐dinitrophenol and of freeze‐thawing disrupted mitochondria were measured. Phosphate release was measured as described in the Materials and Methods section. Each experimental point represented in panel B is the mean ± SEM of 10 independent experiments. (C) Rates of mitochondrial ROS generation were measured as the fluorescence increase due to formation of 2′‐7′‐dichlorofluorescein (DCF), as described in the Materials and Methods section. Each experimental point represented in panel C is the mean ± SEM of 10 independent experiments. (D) The capacity of intact mitochondria in oxidizing exogenously added NADH was evaluated to infer about membrane integrity. Oxygen uptake was taken as an indicator of NADH oxidation. In all assays, EGCG was added to the reaction medium as a dimethylsulfoxide (DMSO) solution. Appropriate controls with DMSO were run in order to exclude eventual solvent effects. Each experimental point represented in panel D is the mean ± SEM of 11 independent experiments. Asterisks indicate statistically significant differences when compared to the controls (*P* ≤ 0.05), as determined by ANOVA followed by Tukey's *post hoc* test.

The possibility of an action of EGCG on the various oxidase activities of mitochondria (Segments I, II and IV) was explored by the experiments shown in Fig. 8. No inhibition was found at concentrations of up to 500 μm. Succinate oxidase and NADH oxidase were mildly inhibited by concentrations above 500 μm, while no inhibition was found at concentrations of up to 1000 μm for the oxidation of TMPD/ascorbate (Complex IV).

State III respiration, which is inhibited by EGCG, is largely controlled by the ATP‐synthase. In order to obtain information about possible direct effects of EGCG on this enzymatic complex, its activity was measured under conditions of ATP hydrolysis. The results are shown in panel B of Fig. 8. In coupled intact mitochondria, where the ATPase activity is very low, there was pronounced stimulation at low EGCG concentrations. This stimulation vanished progressively as the concentration was raised. The same phenomenon occurred when 2,4‐dinitrophenol mitochondria were used: stimulation at low concentrations with a peak shift toward higher concentrations and gradual lessening of stimulation, which turned into inhibition at the highest concentrations. In disrupted mitochondria, however, inhibition occurred practically over the entire concentration range. The observations made herein suggest that the actions of EGCG on the ATP‐synthase‐ATPase are highly structure‐conditioned.

### Free radical generation in isolated mitochondria

The antioxidant action of EGCG has been quantified mainly in chemical systems and less in biological ones. Since mitochondria are possibly the most important site of free radical generation in the cell [33, 40], it is of interest to know how EGCG affects this activity. The results of this experimental series are shown in panel C of Fig. 8. As described in the Materials and Methods section, the continuous free radical generation in consequence of succinate oxidation in the presence of rotenone was measured using a fluorescent indicator. EGCG did not inhibit free radical production at concentrations of up to 5 μm. Free radical quenching started at the concentration of 10 μm but a true expansion of this effect was seen at the concentrations of 20–100 μm.

### Membrane integrity in mitochondria and liver cells

Membrane integrity is essential for many biological phenomena, including intracellular ion gradients (protons) and ATP compartmentation. For this reason, experiments were done in which indicators of membrane integrity were analyzed. It is widely accepted that the mitochondrial membrane is not normally permeable to NAD^+^/NADH [41]. Consequently, the oxidation of NADH by intact mitochondria can be regarded as an indicator of the integrity of their membranes. Panel D in Fig. 8 shows the effects of EGCG on the oxidation of exogenously added NADH. In the absence of EGCG, the rate of NADH oxidation is very low, a situation consistent with a NADH‐impermeable mitochondrial membrane. With increasing EGCG concentrations in the reaction medium, however, the oxidation of NADH increased, reaching a maximum at 500 μm. These results indicate that EGCG impairs the membrane integrity of mitochondria, a phenomenon that might influence compartmentation not only of NAD^+^‐NADH couple but also of other important agents, such as ADP and ATP.

**Fig. 9:**
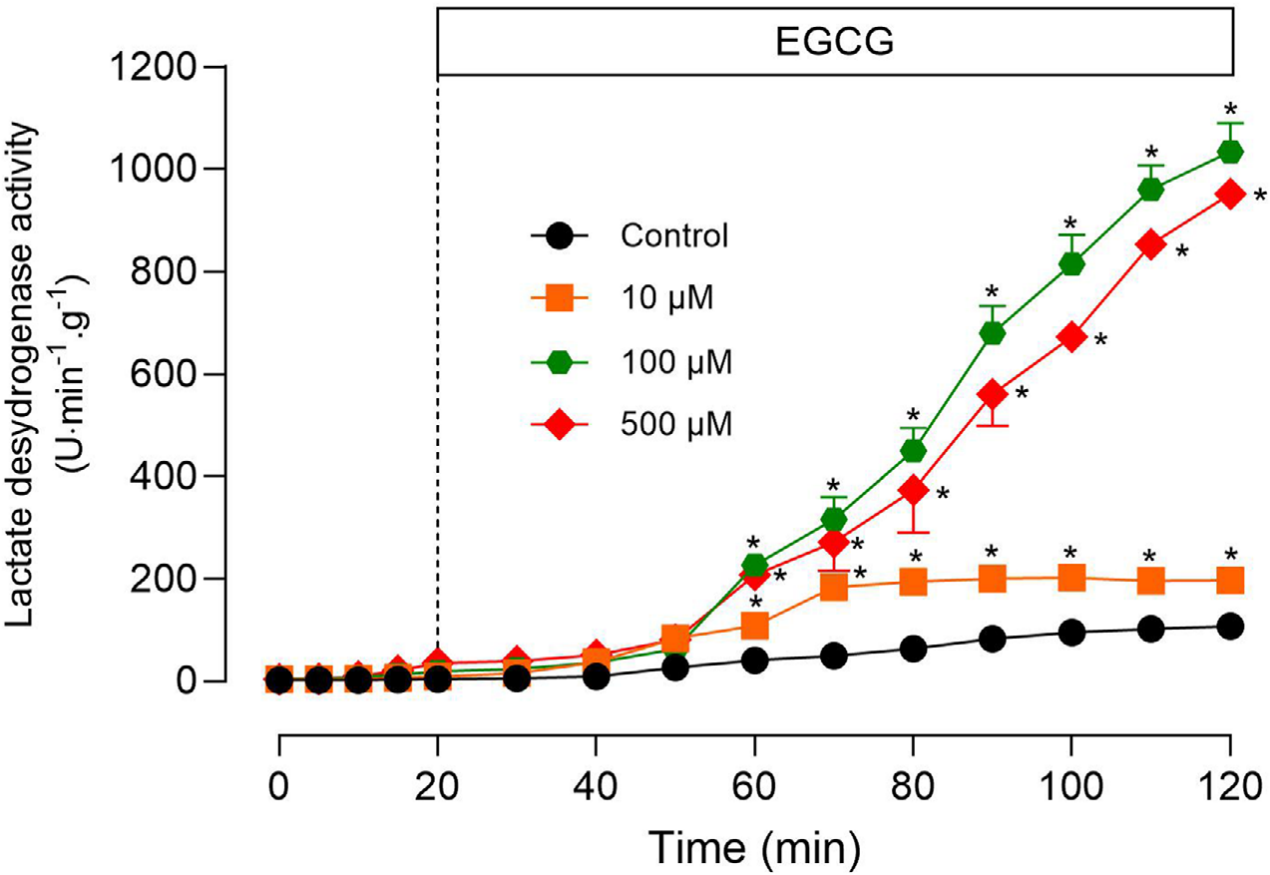
Influence of epigallocatechin‐3‐gallate (EGCG) on the release of lactate dehydrogenase by the perfused liver. Livers from fed rats were perfused with substrate‐free Krebs/Henseleit‐bicarbonate buffer, as described in the Materials and Methods section. After a 20‐min stabilization period, as inferred from oxygen consumption rates, EGCG was infused at various concentrations during 100 min. The effluent perfusate was sampled at 2‐min intervals for assaying the lactate dehydrogenase activity. Values represent means ± SEM. The number of biologically independent experiments for each concentration was: *n* = 5, *n* = 3, *n* = 4, and *n* = 4 for the concentrations 0 (control), 10, 100, and 500 μm, respectively. Asterisks indicate statistically significant differences when compared to the controls (*P* ≤ 0.05), as determined by ANOVA followed by Tukey's *post hoc* test.

Since membranes of different parts of the cell are quite similar concerning their basic structure, it is worth investigating whether the plasma membrane is also damaged by EGCG. Integrity of the hepatocyte membrane can be investigated by measuring the release of macromolecules, such as the highly abundant enzyme l‐lactate dehydrogenase [42]. The results of the experiments are shown in Fig. 9. Livers from fed rats were perfused with substrate‐free perfusion fluid and the infusion of EGCG was started after oxygen uptake stabilization. Three different concentrations were infused, namely 10, 100, and 500 μm. Leakage of l‐lactate dehydrogenase above the control levels was accelerated by EGCG even at the lowest concentration (10 μm). The effect stabilized after 50‐min infusion. With the higher concentrations of 100 and 500 μm, however, the enzyme leakage accelerated continuously up to the end of the perfusion time. These results indicate potential toxic effects, with metabolic consequences that go in the same direction as those detected in mitochondria.

### Effects of EGCG on the hepatic adenine nucleotide contents

**Fig. 10:**
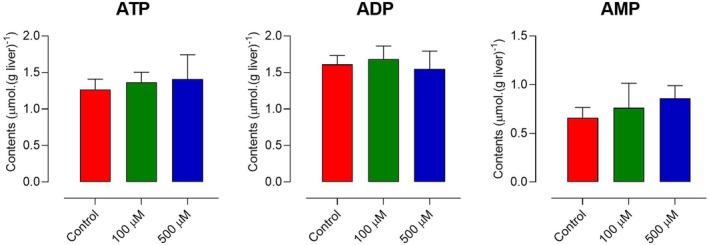
Epigallocatechin‐3‐gallate (EGCG) and the hepatic content of adenine mononucleotides. Livers of fasted rats were perfused as described in the Materials and Methods section. The first group of livers (controls) were perfused with 2 mm lactate alone for 30 min before freeze‐clamping with liquid nitrogen. The second group was perfused with 2 mm during the first 30 min and thereafter cumulatively with 500 μm EGCG for an additional 30 min before the freeze‐clamping procedure. The freeze‐clamped livers were immediately extracted with ice‐cold perchloric acid. After neutralization, the samples were used for the determination of the AMP, ADP, and ATP contents, as described in the Materials and Methods section. Values are means ± SEM (*n* = 6) of biologically independent experiments.

The results of the experiments with isolated mitochondria in overall and, specifically, the diminution of oxygen consumption in the perfused liver that occurred in parallel with the reduction in glucose production, suggest that the cellular levels of ATP might have been reduced by EGCG. For this reason, the adenine mononucleotide contents of the liver cells were quantified. The results are shown in Fig. 10. The assays were conducted under gluconeogenic conditions with lactate as the precursor in an attempt at establishing a correlation between oxygen consumption inhibition and gluconeogenesis inhibition. The results, however, were negative in this respect. EGCG, at the concentrations of 100 as well as of 500 μm did not change the total content of ATP, ADP, or AMP of the liver. There are several possible explanations for this apparent discrepancy, as will be discussed in the following section, but it must be anticipated that the levels that were measured refer to the total contents of the adenine mononucleotides, which in the normally working cell are not uniformly distributed between cytosol and mitochondria [43].

### Effects of EGCG on the activity of enzymes of the gluconeogenic pathway

**Fig. 11:**
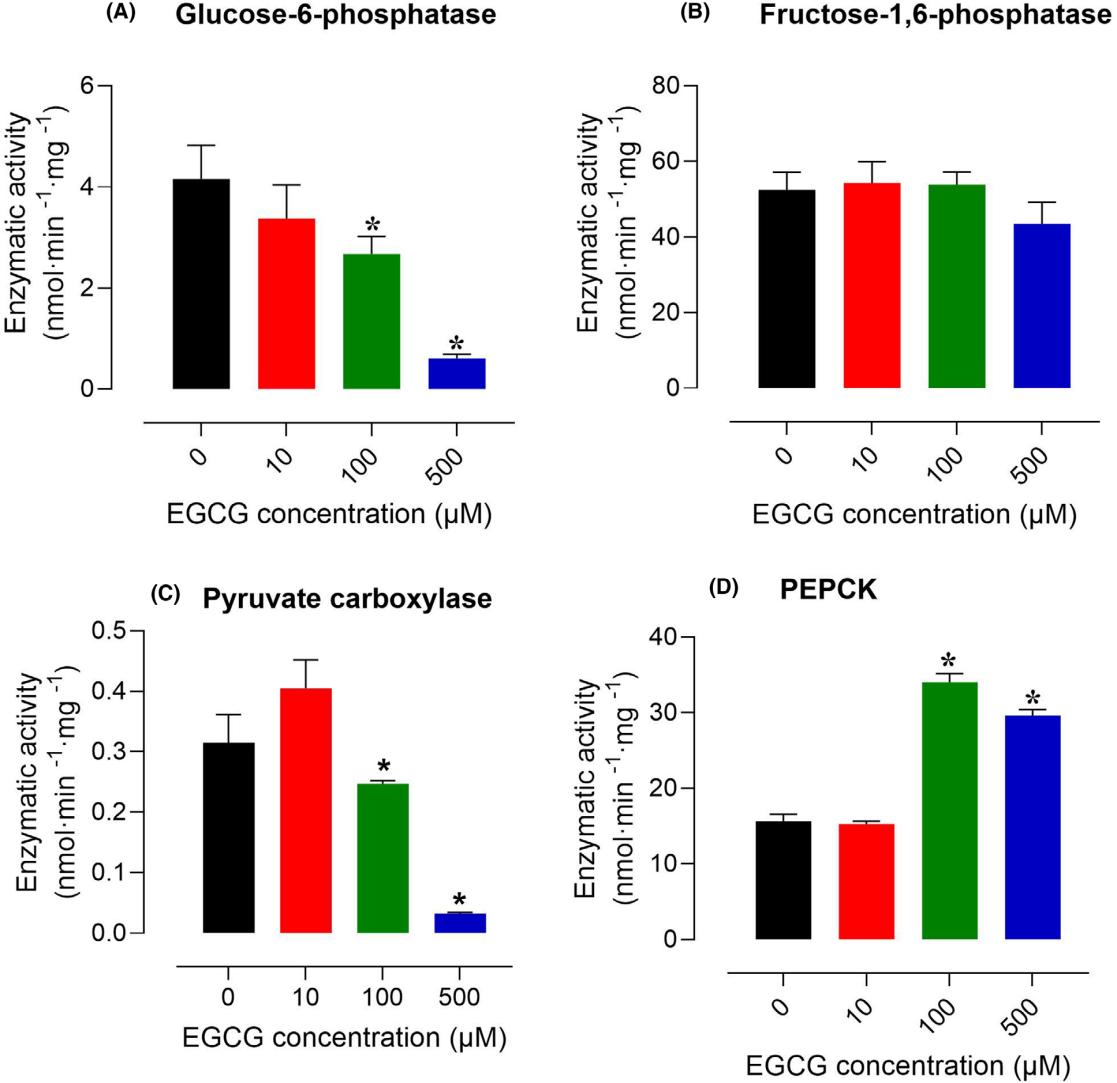
Effects of epigallocatechin‐3‐gallate (EGCG) on enzymes of the gluconeogenic pathway: (A) glucose‐6‐phosphatase; (B) fructose‐1,6‐bisphosphatase; (C) pyruvate carboxylase; (D) phosphoenolpyruvate carboxykinase. In all assays, EGCG was added to the reaction medium as a dimethylsulfoxide (DMSO) solution. Controls were run in order to exclude solvent effects. Each bar represents means ± SEM (*n* = 5) of biologically independent experiments. Asterisks indicate statistically significant differences when compared to the controls (*P* ≤ 0.05), as determined by ANOVA followed by Tukey's *post hoc* test.

Inhibition of enzymes of any metabolic route can also be inhibitory for the pathway as a whole depending on the degree of the inhibition and on the position of the enzyme along the sequence. In the present work, four enzymes of the gluconeogenic pathway were assayed for their sensitivity to EGCG, namely glucose‐6‐phosphatase, fructose‐1,6‐bisphosphatase, phosphoenolpyruvate carboxykinase (PEPCK) and pyruvate carboxylase. The results are shown in Fig. 11. Glucose 6‐phosphatase was inhibited in a concentration‐dependent manner, reaching 83% at the concentration of 500 μm (panel A). The fructose‐1,6‐bisphosphatase, by contrast, was not affected at all by EGCG (panel B).

The pyruvate carboxylase, which catalyzes the step with the highest control strength in the pathway between pyruvate and glucose [44], was inhibited by 100 and 500 μm EGCG, but presented a tendency toward stimulation at 10 μm EGCG (panel C). The phosphoenolpyruvate carboxykinase (PEPCK), by contrast, was stimulated by EGCG (panel D). It must be mentioned, however, that the control strength of this enzyme is minimal when compared to that of other steps as will be discussed in the next section [44].

## Discussion

The effects of EGCG on the liver in general and on the liver metabolism in particular are complex and multifaceted and sometimes even contradictory at first glance. However, the main results, which are inhibition of gluconeogenesis and stimulation of glycolysis (from endogenous glycogen), are phenomena that fit quite well within the current notion that the compound displays antidiabetic properties. In this respect, evidence has been presented elsewhere that EGCG reduces the expression of the genes of glucose‐6‐phosphatase and phosphoenolpyruvate carboxykinase (PEPCK) [4] and increases the expression of the glucokinase gene [5], phenomena that have been appointed exactly as possible mechanisms that can lead to inhibition of gluconeogenesis and stimulation of glycolysis. However, gene expressions are long‐term or at least medium‐term effects, whereas the findings reported herein are clearly short‐term effects that take minutes to manifest themselves. They represent, thus, direct effects, resulting from interactions with enzymes and membranes. But, as mentioned above, they are quite complex and require careful analyses before arriving at meaningful conclusions.

Since several associated parameters were measured, results of these measurements will allow us to devise at least the most important reasons for the most prominent effect, which is gluconeogenesis inhibition. Glucose production from lactate and alanine encompasses the complete pathway, which is compartmentalized between mitochondria and cytosol. The analysis that follows is centered on the effects of EGCG on the enzymes of the pathway. This will be done taking into account their flux control coefficients, which are available in the literature thanks to the work of Groen *et al*. [44]. These authors determined the flux control coefficients in hepatocytes under perfusion with lactate as the gluconeogenic substrate, present at three different concentrations: 0.5, 1.0, and 5.0 mm. The availability of the flux control coefficients is very important because it allows us to discuss the influence of inhibitory or stimulatory effects on enzymatic steps along a given pathway in quantitative terms rather than speculating in mere qualitative terms. The values that will be used here were obtained by numerical interpolation of the concentration used in the present work (2 mm) into the original concentration dependences published by Groen *et al*. [44]. EGCG inhibited pyruvate carboxylation, which is catalyzed by the mitochondrial pyruvate carboxylase (PC):

(1) $$\text{Pyruvate}+{\mathrm{CO}}_{2}+\mathrm{ATP}\;\overset{\mathrm{PC}}{\to }\;\text{oxaloacetate}+\mathrm{ADP}+P_{i}$$

It was found in our experiments that EGCG inhibits this enzyme either directly or via inhibition of pyruvate transport across the mitochondrial membrane. What gluconeogenesis from lactate and also from alanine concerns, this is possibly the most important event behind the inhibition of gluconeogenesis by the simple fact that the flux control coefficient of PC is equal to 66.9%. This is a very high value if one considers that the algebraic sum of all flux control coefficients in a given metabolic pathway is equal to 100% [44]. The flux control coefficient of pyruvate carboxylase, thus, considerably exceeds that of any other enzyme in the gluconeogenesis pathway.

The enzyme phosphoenolpruvate carboxykinase (PEPCK), which is situated downstream of pyruvate carboxylase, is frequently mentioned as a key enzyme of gluconeogenesis, especially in gene expression studies:

(2) $$\begin{array}{l} \text{Oxaloacetate}+\mathrm{GTP}\overset{\text{PEPCK}}{\to }\text{phosphoenolpyruvate}\\ \;+\;\mathrm{GDP}+{\mathrm{CO}}_{2} \end{array}$$

However, its flux control coefficient is only 1.95%. It can eventually become a rate‐limiting step if its activity drops to an appreciable extent by drastically diminished gene expression or by strong inhibition. On the contrary, PEPCK was stimulated by EGCG, an observation that disqualifies it as a site for gluconeogenesis inhibition. Possibly, the control coefficient of PEPCK is even diminished by EGCG.

Another important point of control of gluconeogenesis in which EGCG is involved, this time in an indirect way, is the gluconeogenesis pathway segment which goes from the glyceraldehyde 3‐phosphate dehydrogenase reaction (GAPDH) to the enolase reaction. In their attempts at quantifying the flux control coefficients in gluconeogenesis, Groen *et al*. [45] lumped together all the enzymes of this segment:

(3) $$\begin{array}{l} \text{Phosphoenolpyruvate}+H_{2}O\overset{\text{Enolase}}{⇄}2‐\text{phosphoglycerate}\overset{\mathrm{PGM}}{⇄}3‐\text{phosphoglycerate}\\ 3‐\text{Phosphoglycerate}+\mathrm{ATP}\overset{\mathrm{PGK}}{⇄}1,3‐\text{bisphosphoglycerate}+\mathrm{ADP}1\\ 1,3‐\text{Bisphosphoglycerate}+\text{NADH}+H^{+}\overset{\text{GAPDH}}{⇄}P_{i}+\;{\mathrm{NAD}}^{+}+\text{glyceraldehyde}\;3‐\text{phosphate} \end{array}$$

The first two, enolase and phosphoglycerate mutase (PGM), are equilibrium enzymes, which are unlikely to exert significant control. The 3‐phosphoglycerate kinase (PGK) and the GAPDH display together, however, a flux control coefficient of 17.6%, which is highly significant. GAPDH is the enzyme that catalyzes the step at which reducing power, which is needed for glucose synthesis, is effectively used in the form of NADH. The importance of reducing power in the form of NADH has been emphasized in several pioneer studies [46]. There are at least two kinds of observations in the present work indicating that the cellular concentrations of NADH were diminished by EGCG. Diminutions of the NADH/NAD^+^ ratio by EGCG were found when measuring lactate and pyruvate productions in three metabolic pathways: glycerol and alanine metabolism and glycogen catabolism. It is amply accepted that the lactate to pyruvate ratio reflects the cytosolic NADH/NAD^+^ ratio due to the lactate dehydrogenase equilibrium [37]. Furthermore, the phenomenon also extends to the mitochondria, as indicated by the diminution of the β‐hydroxybutyrate/acetoacetate ratio, an indicator of the NADH/NAD^+^ ratio in the mitochondria [37]. All these observations suggest, thus, that EGCG diminishes the NADH but increases the NAD^+^ concentration in the cytosol, a situation that should disfavor the GAPDH reaction in the direction of glucose synthesis. It seems, thus, a valid conclusion that the diminished NADH/NAD^+^ ratios caused by EGCG could be another cause for its inhibitory action on gluconeogenesis. This is also probably the main reason for the observed inhibition on gluconeogenesis from glycerol. Supporting this hypothesis is the observation that inhibition of glucose production from glycerol is accompanied by a substantial increase in lactate production and an even more pronounced increase in pyruvate production.

The decreased NADH/NAD^+^ ratio under the influence of EGCG can be the consequence of the facilitated oxidation of cytosolic NADH in the mitochondria, which the compound induces in these organelles by increasing membrane permeability, although this may not be the sole mechanism. The phenomenon occurs at low EGCG concentrations and is probably analogous to the increased cell membrane permeability revealed by the leaking of lactate dehydrogenase. Protein losses are usually considered indicators for changes in membrane fluidity, trafficking, and permeability [47, 48].

The reaction group (3) above also includes a step in which ATP participates. Actually, ATP is also required in reactions (1) and (2). In general, conditions in which the cytosolic or mitochondrial content of this nucleotide is reduced also result in diminished gluconeogenesis [49, 50]. Measurements performed in the present study, however, did not reveal significant changes in the total hepatic contents of AMP, ADP, or ATP during EGCG infusion. [Correction added on 26th September 2025 after first online publication: This paragraph has been modified.]

The group of gluconeogenic enzymes that includes the 1,6‐bisphosphatase has a control coefficient of 10.8%, according to Groen *et al*. [44]:

(4) $$\begin{array}{l} \text{glyceraldehyde}\;3‐P_{i}\overset{\mathrm{TIM}}{⇄}\text{dihydroxyacetone}‐P_{i}\overset{\text{Aldolase}}{\to }\text{fructose}‐1,6‐\text{bisphosphate}\\ \text{Fructose}‐1,6‐\text{bisphosphate}+H_{2}O\overset{\text{Fructose}\;1,6-\text{bisphosphatase}}{\to }\text{fructose}\;6‐\text{phosphate}+\;P_{i} \end{array}$$

A flux control coefficient of 10.8% can be significant, but our measurements failed to detect direct inhibition of fructose 1,6‐bisphosphatase. The latter is a regulatory enzyme, but no information about this possibility was obtained in the experiments of the present work. On the contrary, EGCG inhibited glucose 6‐phosphatase, the last enzyme of the gluconeogenic pathway:

(5) $$\begin{array}{l} \text{Fructose}\;6‐\text{phosphate}\overset{\text{Phosphogluco}\text{isomerase}}{⇄}\text{glucose}\;6‐\text{phosphate}\\ \text{Glucose}\;6‐\text{phosphate}+H_{2}O\;\overset{\mathrm{Glu}\cos e\;6‐\text{phosphatase}}{\to }\;\text{glucose}+P_{i} \end{array}$$

The flux control coefficient of group (5), however, was found to be equal to 1.1%. Only very high degrees of inhibition, thus, will be able to affect glucose output in the liver, as indeed demonstrated with the inhibitor isosteviol [51]. This is because the primary consequence when a terminal enzyme of a given pathway is inhibited is that the substrate concentration increases in order to restore the previous steady‐state flux. This occurs unless the enzyme is already saturated, but the glucose 6‐phosphatase has a high K_M_ compared with the usual cellular glucose 6‐phosphate concentrations [12]. An increased cellular glucose 6‐phosphate concentration, however, may have consequences for other pathways, such as glycolysis from endogenous glycogen and also for gluconeogenesis precursors that enter the pathway at the level of the glyceraldehyde 3‐phosphate dehydrogenase, as is the case of glycerol.

The increased glycolytic flux observed with EGCG is likely also related to the decreased NADH/NAD^+^ ratio [37]. In the absence of exogenous glucose, this enhanced glycolysis depends on glucosyl units derived from endogenous glycogen stores. However, the direct involvement of glycogen phosphorylase (GP) in this effect requires careful consideration. Recent evidence indicates that EGCG can directly inhibit GP activity. Alexopoulos *et al*. [52] demonstrated that EGCG binds to and suppresses liver GP activity *in vitro*, suggesting that its action on glycogenolysis may not result from direct activation of the enzyme, but rather from indirect regulatory mechanisms or compensatory shifts in metabolic fluxes. In our model, the net increase in glycolytic flux despite this possible inhibitory effect on GP may be explained by EGCG‐induced changes in the redox balance—specifically, a more oxidized cytosolic state that favors the glyceraldehyde‐3‐phosphate dehydrogenase (GAPDH) reaction—and by elevated glucose‐6‐phosphate levels arising from other pathways of glycogen mobilization. These combined effects could override any partial inhibition of GP, ultimately sustaining increased glycolysis under the experimental conditions.

There is an apparent contradiction with respect to the effects of EGCG on oxygen consumption as stimulation or inhibition occurred under different conditions. However, this apparent contradiction has a logical explanation based on current knowledge about the physiology of the liver cells. Stimulation of oxygen uptake, which occurred in the livers with active glycogen catabolism and oleic acid oxidation is actually the natural and expected effect for several reasons. EGCG causes mild uncoupling, as demonstrated in experiments with isolated mitochondria at concentrations of up to 500 μm. Mild uncoupling can be defined as a situation in which there is mainly stimulation of mitochondrial oxygen consumption without, however, significant impairment of ATP synthesis [50, 53]. In fact, no diminution in the ATP content in the liver was found at concentrations of up to 500 μm. The increase in oxygen uptake caused by EGCG when it was infused in the presence of oleic acid was not stable. This suggests that the oxidation of oleic acid was gradually being replaced in part by endogenous fatty acids in adaptation to the new conditions caused by the introduction of EGCG.

In addition to mild uncoupling, another cause for oxygen consumption stimulation by EGCG is the apparent abolition caused by this compound of the control exerted by the shuttle systems in the oxidation of NADH. The increased permeability of the mitochondrial membrane to NADH strongly suggests that the cytosolic and mitochondrial pools practically merge into a single enlarged pool, greatly facilitating the access of the cytosolic reducing equivalents to the respiratory chain.

The inhibition of oxygen consumption by EGCG that occurred upon the introduction of lactate, alanine, and glycerol in livers from fasted rats was in all cases preceded by a transient increase in oxygen consumption. It should be recalled, however, that oxygen consumption had been previously stimulated during the infusion of those substrates for the stringent reason that gluconeogenesis is a phenomenon that requires a considerable amount of energy in the form of ATP (6 mol ATP or equivalents per mol glucose). EGCG, as discussed above, inhibits gluconeogenesis for reasons that are not directly related to the production of ATP in the mitochondria. The diminution in oxygen uptake under gluconeogenic conditions reflects, thus, most probably, the reduced needs of energy in the presence of EGCG. In the case of glycerol, inhibition of oxygen uptake surpassed the previous levels that were observed before the infusion of this substrate, a phenomenon that did not occur when the substrates were lactate and alanine. The cause of this phenomenon may be linked to the fact that glycerol transformation in the glycolytic pathway, which was stimulated by EGCG, caused additional ATP production, a phenomenon that further diminished the need for mitochondrial ATP.

Considering the relevance and implications of the observed effects, it is important to highlight that EGCG can be ingested orally in the form of capsules and food supplements prepared from highly concentrated *Camellia sinensis* extracts or in the form of relatively diluted infusions (tea). The blood concentration of EGCG after tea consumption is very low (0.3 to 0.5 μm) [6]. The ingestion of 50 to 1600 mg EGCG under fasting conditions by healthy volunteers may result in plasma concentrations of 0.3 to 7 μm [54]. This plasma concentration is smaller than the concentrations that resulted in the full development of the metabolic transformations described in the present work, except perhaps for the effects on the mitochondrial membranes and the mild inhibitory actions on gluconeogenesis. However, the plasma concentration for a drug that enters the organism via the portal vein does not reflect its concentration in this vessel. EGCG is extensively metabolized in the liver, where it undergoes glucuronidation by UDP‐glucuronosyltransferase, sulfation by sulfotransferase, and methylation by catechol‐O‐methyltransferase [54]. The portal concentration of drugs that are extensively metabolized in the liver is usually much higher than systemic concentrations by factors varying between 10 and 1000 (depending on time and nature of the compound), as it was already determined by studies using a portal vein cannulation technique [55, 56]. Furthermore, most effects observed in the perfused liver are not reversed shortly after EGCG is withdrawn, suggesting that the drug, or at least its effects, tend to remain for relatively long periods.

For the reasons discussed above, the hypothesis can be raised that the direct effects of EGCG in the liver that were observed herein may represent a relevant contribution to the *in vivo* reported effects of the compound, especially the antihyperglycemic ones. The abnormal increase in the hepatic gluconeogenesis is a significant event in patients with type 2 diabetes mellitus due to insulin resistance [57]. The capacity of EGCG in reducing directly hepatic gluconeogenesis from various substrates can, thus, be regarded as a short‐term mechanism related to its antidiabetic activity, which has not yet been reported before.

Another relevant observation of the present work is the direct stimulation of the oxidation of fatty acids in the liver, which could bear relation to the reported antiobesity action of EGCG [58]. These results are in agreement with the studies that demonstrated for EGCG the ability to inhibit the accumulation of lipids in adipocytes and hepatocytes *in vitro* through inhibition of synthesis and stimulation of fatty acid oxidation. It has been suggested that these effects are related to the protein kinase pathway activated by AMP (AMPK), which in turn can be activated by a redox regulated mechanism induced by increased ROS levels in the cells [59, 60]. It is true that our results showed that EGCG is capable of impairing ROS production in mitochondria, but at the same time, the compound was capable of shifting the redox state of the liver to a more oxidized state in both cytosol and mitochondria. The latter phenomenon, in addition to its possible influence on glycolysis and gluconeogenesis, can also affect the antioxidant capacity of the liver, resulting in increased ROS and consequent activation of AMPK.

Our results also suggest that, partly at least, the metabolic effects are linked to structural lesions to the hepatocytes, especially in the cellular membranes domain. Shifting of the redox state, for example, may be related to the observed increases in membrane permeability and trafficking, especially in the mitochondria. These phenomena, which can be accompanied by increases in membrane fluidity, may not be exceedingly harmful in the normal and healthy liver cells. However, they may become more pernicious when their intensity increases or in cases where the liver cell membranes and other structures are already weakened, as it happens, for example, in hepatic steatosis (fatty liver disease). Hepatotoxic reactions caused by EGCG have already been reported [61], though the mechanisms are not yet fully understood. All these possibilities highlight the importance of more studies related to the safety of EGCG ingestion, especially at the highest doses and by individuals that might suffer, even mildly, from certain liver disturbances.

In general, the observations of this work allow us to conclude that EGCG is capable of acting directly on several metabolic fluxes in the liver. The compound inhibits gluconeogenesis from at least three substrates, namely lactate, glycerol, and alanine. Conversely, it can stimulate glycolysis, glycogenolysis, and the oxidation of oleic acid. Oxygen consumption can be inhibited or stimulated, depending on the metabolic state of the liver. The mechanisms behind these modifications are complex and diverse. EGCG can act as a mild uncoupler in mitochondria and inhibit enzymes of the gluconeogenic pathway, such as glucose 6‐phosphatase and pyruvate carboxylase. It impairs membrane integrity, a phenomenon that leads to modifications in permeability and cellular trafficking. The latter behavior could be responsible for the pronounced shift in the NADH‐NAD^+^ redox potential toward a more oxidized state. It can be concluded that inhibition of gluconeogenesis might be contributing to the reported antidiabetic effects of EGCG; stimulation of fatty acid oxidation, in turn, could be linked to the reported antiobesity actions. However, the impairment of membrane integrity can be regarded as a potentially toxic manifestation that needs clarification, especially in the case of pathological conditions where both membrane integrity and function are already impaired to some degree.

## Conflict of interest

The authors declare no conflict of interest.

## Author contributions

CIB performed the experiments and analyzed the data. BLC, FCNM, VOP, and NMQEM performed the experiments. AB interpreted the data and wrote the manuscript. JFC and ABSN critically revised the manuscript and provided funding and resources for the research project. LB conceived and designed the study, performed the experiments, and wrote the manuscript. All authors have read and approved the final manuscript.

## Data Availability

The data that support the findings of this study are available in Zenodo at https://doi.org/10.5281/zenodo.15350488, Bracht, L. (2025). Data article Bonetti *et al*. [Data set]. Zenodo. https://doi.org/10.5281/zenodo.15350488.
